# Therapy Dogs in Educational Settings: Guidelines and Recommendations for Implementation

**DOI:** 10.3389/fvets.2021.655104

**Published:** 2021-06-08

**Authors:** Christine Grové, Linda Henderson, Felicia Lee, Phoebe Wardlaw

**Affiliations:** Faculty of Education, Monash University, Victoria, VIC, Australia

**Keywords:** therapy dogs, education, psychology, guidelines and recommendations, school community

## Abstract

Therapy dogs in educational settings have gained increase traction in recent years. Despite its potential benefits and popularity, there remain concerns of perceived risks such as sanitation, allergies, and safety issues, as well as a lack of guidelines, regulations and support resources available to school staff. Research is further lacking into the implementation process of therapy dog programs in educational settings. To construct a set of recommendations for school staff to achieve successful implementation of a therapy dog program, the present study investigated the perceived facilitative and impeding factors when implementing a therapy dog program. A total of 13 school staff and 2 coordinators from therapy dog organisations took part in an open-ended online survey and/or a semi-structured interview over the phone, with the aim of gathering their perspectives of implementing a therapy dog program in schools. The thematic analysis of the data indicated facilitative factors such as program flexibility, whole-school support, the need for communication and training for all staff, as well as dog welfare. Successful implementation of therapy dogs in an education setting appear to revolve around (1) flexibility of the dog therapy program to target school's needs, (2) qualities of program instigator, (3) whole-school support, (4) communication, training and education, (5) considerations for dog's welfare. Key barriers identified included a high workload, lack of guidelines on processes, lack of support from the school community, as well as the need for better understanding of the role of a therapy dog. The results highlighted the importance of a whole-school effort when implementing a therapy dog program, as well as the need for guidelines for assessing school readiness, key factors for consideration, and strategies to overcome challenges associated with program implementation.

## Introduction

### Therapy Dogs and Therapy Dog Organisations

Therapy dogs in educational settings have gained traction in recent times. Increasingly, dogs have provided therapeutic support in early education settings, primary and secondary schools, as well as university settings to support students' well-being, promote a sense of belonging in school, reduce stress and anxiety, and even facilitate learning (1–3). Across the globe, the incorporation of a therapy dog as part of a school programs and activities such as dog-assisted reading programs, has increased exponentially in prevalence. For example, therapy dog organisations in Australia which conduct dog-assisted reading programs now include Story Dogs which originated in New South Wales, Delta Classroom Canines™ from Victoria, and Special Human Animal Relationships in Education (S.H.A.R.E. Reading Dogs program) from Gold Coast. Story Dogs, for example, presently partners with 247 schools across Australia and works with over 2,100 children each week (1, 4).

Several terms describe how dogs provide therapeutic benefit. A therapy dog refers to a dog trained to provide therapeutic benefit mainly through Animal-Assisted Therapy (AAT), Animal-Assisted Intervention (AAI), or Animal-Assisted Education (AAE) (2). AAI, Animal Assisted Interventions, is an umbrella term that encompasses AAT, AAE, Animal Assisted Activity (AAA) and Animal Assisted Counselling (AAC) (5). The dog themselves do not provide therapy, but rather how they are engaged and connected with in the program is what may be therapeutic. The authors use the term “therapy dog” to attempt to differentiate between trained dogs for a purpose vs. dogs that have not undertaken training or are not accredited dogs. Therapy dogs are not therapists. Work with a dog may be therapeutic or it may be an activity such coaching or assisted reading where the dog's role is to be present but not necessarily actively engaged.

Therapy dogs are first selected for their temperament and subsequently trained together with their handler, usually by therapy dog organisations, to be calm and obedient such that there are able to provide emotional support, comfort, and companionship to different individuals in various educational, health care, and community settings (2). In AAT, the therapy dog forms part of a professional therapeutic treatment with a professional to deliver a specialised expertise and practise based on the professional's profession (2). For example, assisting a psychologist to provide regular, structured, and tailored intervention for a client with mental health concerns. In AAA, the relationship is more casual and typically designed to achieve broader goals and handled by volunteers who may not have specific professional training to provide interventions (6, 7). An example of a therapy dog being involved in AAI is providing general comfort and companionship to patients at a hospital or to students at a university before an examination. Lastly in AAE, the therapy dog is in an educational setting to facilitate in the learning and education of students, for example, through dog-assisted reading programs. Therapy dogs are distinctively different from service or guide dogs, with the latter trained for the purpose of meeting the specific needs of one individual in the long term. For example, a service dog assisting a child with epilepsy is specifically trained to identify signs of a seizure, or a guide dog supporting an individual who is visually impaired to be independent in daily functioning.

### Impact of Therapy Dogs on Children's Well-Being and Learning

There is preliminary evidence which suggests that therapy dogs can enhance children's well-being in a variety of settings from schools, hospitals, airports, and courtrooms. Therapy dogs have been found to reduce physiological symptoms of stress through lowering cortisol levels (8), increasing positive emotions (1, 9–13), promoting engagement in learning activities and positive attitudes toward learning (6, 11, 14, 15), reducing negative behaviours like task avoidance and aggression in the classroom (16–19), as well as encouraging prosocial behaviours and acting as a “social catalyst” to facilitate social interactions with others (16–18, 20, 21).

In addition, there is evidence indicating an association between well-being and learning outcomes (22–25). Children with higher levels of well-being learn more effectively, have lower levels of absenteeism at school, better academic engagement, and also have more satisfying and successful peer relationships (24, 25). There is also an association between children's well-being and reading outcomes (22, 23). Research on therapy dogs involved in facilitating well-being and learning outcomes such as reading through dog-assisted reading programs have found some promising results, although there are methodological limitations in some studies. For example, challenges in associating improvements to the therapy dog vs. other factors such as the program or person implementing the program. Despite these limitations, a systematic literature review by Hall et al. (26) found that dog-assisted reading programs generally show promising results such as gains in reading skills (e.g., reading accuracy, oral reading fluency, comprehension), as well as more positive attitudes and improved behaviours toward reading. While further research is warranted, researchers have proposed that participation in a therapy dog program like dog-assisted programs can lead to improvements in emotional and behavioural processes (e.g., reduced anxiety, increased self-esteem, enhanced motivation and hence, overall well-being), which in turn can facilitate learning and contribute to gains in learning outcomes like reading (26).

### Concerns, Risks, and Objections

Despite the potential benefits of a therapy dog program, there are several concerns and challenges which might arise from successfully introducing a therapy dog into a school context. Primary concerns by schools include legal implications and liability, allergy concerns, hygiene and sanitation concerns, safety concerns, cultural differences, fear of dogs, animal welfare, funding associated with animal maintenance and program implementation, as well as a lack of administrative support (2, 27–32).

The most common objection for introducing a therapy dog into a school setting is sanitation concerns. There is a common perception that dogs can be potential carriers of diseases and infections which can then be transmitted to human beings (30). Allergies due to animal dander, the most common source of allergic reactions, as well as safety concerns around dog bites in children and perceptions of dogs being fierce, aggressive, or protective in nature, are also major deterrents to approving the incorporation of a therapy dog in schools (28, 30). Some cultures also regard dogs as unsanitary (28, 30). In addition, there are ethical issues surrounding animal welfare such as ensuring that the therapy dog's needs are met in the school setting, is safe, and is not overworked (33).

Another barrier to implementing therapy dogs and programs into schools is funding as well as a lack of administrative and staff support. There are substantial costs involving a therapy dog. In addition to the usual costs associated with owning a dog, the costs associated with annual veterinary check-ups, vaccinations, as well as therapy dog training can be quite substantial (32). Most therapy dog work is voluntary (32). In school settings, it is usually the school counsellor, psychologist, teacher or principal who acts as both the owner and handler of the school's therapy dog (32). The handler usually takes on additional duties associated with the therapy dog on top of their usual duties. It has been found that a high staff workload is a negative factor of therapy dog program implementation across multiple settings (34, 35). Often, the handler is responsible to feed, water, walk, groom, and care for the dog, in addition to planning and running all aspects of the therapy dog program independently (36). A lack of knowledge as well as resistance amongst other staff due to various reasons during the phases of therapy dog planning and implementation is also another challenge which handlers need to navigate. Overall, staff burnout, staffing and workload concerns, as well as staff attitudes toward a therapy dog program are key factors which require consideration before implementing a therapy dog program as they can have a significant negative effect on the uptake of therapy dog programs into multiple facilities (34, 37). The preceding objections and concerns thus need to be addressed in future research and recognised as barriers to implementing programs in schools, in an effort for children to reap the potential benefits therapy dogs have to offer.

### Implementation Science in Educational Settings

Implementation science promotes research findings in healthcare, community and policy contexts (38). It seeks to bridge the gap between research findings and applying those findings in real-world settings in a way that optimises positive outcomes (38). Implementation is not a single event but a process. To understand how successful a given implementation process is working, several factors including the acceptability of the program, appropriateness, adoption, feasibility, fidelity, implementation cost, coverage and sustainability need to be considered (39, 40).

A scoping review of program implementation in the education setting identified several key factors for consideration when implementing a program (39). They found that implementation fidelity—the degree to which the intervention has been implemented as intended by its developers—was the most acknowledged and measured outcome for successful implementation of programs in education settings. Training and ongoing support for all stakeholders and participants through the provision of single or multiple days of workshop activities, as well as the provision of additional resources (e.g., support from experts, virtual technology) was also seen as a dominant strategy (39).

In addition, support for front-line staff was paramount for implementation success as educators often struggle when trying to apply the new practises in the classroom (39). Support provided through continuous feedback, supervision, coaching, and practise observation is critical in achieving successful implementation (41–47). Past research has also indicated the importance of leadership for the successful uptake of interventions into multiple settings (48–50). Aarons et al. (48) highlighted qualities such as creating a vision for the uptake of evidence-based interventions in the school or educational system, engaging faculty and other staff in this vision and its realisation, as well as being a role model in realising the vision. Displaying these leadership qualities have been found to provide front-line staff with clear expectations (39).

### Guidelines and Regulations for Program Implementation

Given the concerns and challenges associated with implementing a therapy dog program in schools, as well as the various factors for consideration when implementing any new practise, it is critical that schools have guidelines on key considerations and processes to help them navigate this implementation process. There are currently few resources available for accessing guidelines, procedures and standards for implementing a therapy dog program into various settings and populations (51–54). For example, Fine (52) created guidelines and best practises for using dogs as therapeutic companions with multiple populations, including children, in therapeutic settings. He also included animal selection criteria, animal welfare, training and certification procedures, as well as ways to introduce a therapy dog to clients (52). The Delta Society's Pet Partner Program and Delta Society's (51) Standards of Practise for Animal- Assisted Activities and Therapy also provides guidance in administrative structure, standards of practise, personnel credentials, vocational profiles, treatment plan development, documentation, sample forms, and a bibliography (53). In addition, there are a number of guidelines in other settings such as healthcare facilities (15, 54–57). Despite the wealth of information provided in the above guidelines, there are however no specific and evidence based guidelines on implementing a therapy dog program into a school setting (to the best of the authors knowledge). In addition, guides created by Departments of Education tend to focus on animals for teaching (e.g., science lessons), or assistance animals (e.g., guide dogs), or visiting animals with brief information emphasising the importance of animal welfare, safety, hygiene, and sanitation procedures when interacting with animals in other contexts (e.g., school excursions, classroom pets) rather than extensive guidelines and recommendations for therapy dog program implementation.

### The Present Study

While therapy dog programs have increasingly been incorporated in many school settings, research exploring the specific concerns and challenges associated with implementation in schools are needed. Moreover, guidelines, policies and existing research into therapy dog programs is lacking. The current study examined the following three research questions:

1. What are the facilitative factors associated with implementing a therapy dog program in schools?
2. What are the perceived challenges and barriers with implementing a therapy dog program in schools?
3. What support is required when implementing a therapy dog program in schools?

The results of the study informed a set of research-informed recommendations and guidelines outlining key factors for consideration when implementing a therapy dog program in school settings.

## Method

### Research Design

A qualitative research design was employed using open-ended questionnaires in form of an online survey and/or semi-structured interviews completed over the phone.

### Participants

Data was drawn from two participant groups: (i) schools with an existing therapy dog program, (ii) schools considering implementing a therapy dog program, and (iii) therapy dog organisations. All participants were recruited via email and snowballing. Recruitment for this study began with schools which approached the principal researchers to express their interest in implementing a therapy dog program in their school. Further snowball sampling and comprehensive investigation online were conducted to identify participants in all three groups who were emailed to seek their interest in taking part in the study.

### Qualitative Interviews

Rapport building techniques were used, and a natural conversational tone was maintained by showing empathy, and using active listening skills (Irwin and Johnson, 2005). Probing questions gave school staff the opportunity to elaborate more than others depending on how much information they wished to share. For example, in one interview a participant stated they felt they were “flying-solo,” and the researcher followed with “What do you mean by flying-solo? Can you give me some more information on that experience?” The questionnaire questions (see Appendices A, D) were used as a basis for the semi-structured interviews. All interviews were audio recorded and transcribed with the participants' consent. Participants were labelled by an assigned number (for the purpose of differentiating between participants and to have a chronological record of data collection) as well as their participation method (interview or survey) to maintain confidentiality.

#### Schools

Participants group 1 and 2 consisted of educational staff working in a school setting which included teachers, assistant principals, principals, junior heads of schools, coordinators of early learning centres, school psychologists as heads of well-being and specialist student empowerment teams. Participants were from primary schools, combined primary and secondary schools, and early learning centres in Australia. In total, thirteen school staff across three states in Australia (Victoria, New South Wales and Australian Capital Territory) took part in the study. Participant group 1 consisted of schools with an existing therapy dog program (*N* = 9, 1 male and 8 females) and participant group 2 consisted of schools considering the implementation of a therapy dog program (*N* = 4, 1 male and 3 females).

#### Therapy Dog Organisations

Participant group 3 consisted of staff from two therapy dog organisations, Story Dogs and Delta Therapy Dogs – both non-profit organisations which implement dog-assisted reading programs in schools across Australia. Participants were staff in managerial and coordinator roles (*N* = 2, 2 females). The Story Dogs program is implemented mainly individually where a child reads one-on-one to a dog and handler team for ~20 min every week over at least two school terms. Dog and handlers may also be involved at whole of school assemblies on special occasions such as book week one or twice annually (1). Similarly, for Delta Therapy Dogs, the dog-assisted reading program takes place mainly individually or in small groups where the child or group of children read to a dog and handler team.

The demographic information of all participants is outlined in Table 1.

**Table 1:** Summary of demographic information for participants.

**Pseudonym and participation method**	**Participant group (1/2)**	**Gender**	**Educational institution**	**Designation**	**State in Australia**	**Program type**
Interviewee 1	1	Female	Primary School	Psychologist (Well-being Officer)	VIC	Non-specified
Interviewee 2	1	Female	Primary School	Principal	VIC	Non-specified
Interviewee 3	1	Female	Primary School and Kindergarten	Classroom Teacher	VIC	Non-specified
Interviewee 4	1	Female	Department of Education and Training	Employed DET staff working at schools on program involving therapy dogs	VIC	DET funded pilot program build the capacity of schools to support their disengaging and disengaged students
Interviewee 5	1	Female	Primary School	Classroom Teacher	VIC	PAWS Program
Interviewee 6	2	Male	Primary School	Assistant Principal	VIC	N/A
Interviewee 7	1	Female	Primary School	Psychologist (Student Welfare)	VIC	Non-specified
Interviewee 8	1	Female	Secondary School	Classroom Teacher	VIC	Autism-Assistant Dog for individual student
Interviewee 9	1	Female	Primary School	Learning Specialist/Positive Behaviour Co-Ordinator	VIC	DEPAWs Program
Survey 1	2	Female	Secondary School	Classroom Teacher	VIC	N/A
Survey 2	2	Female	Primary School	Teacher in Learning Support Unit	ACT	N/A
Survey 3	2	Female	Early Learning Centre	Centre Coordinator	VIC	N/A
Survey 4	1	Male	Combined Primary and Secondary School	Head of Junior School	NSW	Non-Specified
Survey 5	3	Female	Therapy Dog Organisation	Managing Director	VIC	Dog-assisted reading program
Survey 6	3	Female	Therapy Dog Organisation	Coordinator	VIC	Dog-assisted reading program

### Materials

A mix of online surveys and semi-structured interviews were conducted with school participants (participant groups 1 and 2). Participants from therapy dog organisations (participant group 3) only completed the online survey. Both methods of data collection—the online survey and semi-structured interviews—involved similar questions. Three separate online surveys were constructed for (i) participants from schools considering a therapy dog program, (ii) participants from schools with an existing therapy dog program, and (iii) participants from therapy dog organisations. The Hexagon Tool developed by the National Implementation Research Network (58) was used to guide the construction of the open-ended surveys. The Hexagon Tool is designed for communities and organisations in any field to evaluate new and existing programs and practises to determine a program's fit within a given context (58). A sample question from the questionnaire for participant group 1 is “Are there any negative outcomes associated with having a therapy dog program in the school? If yes, what are they?” A sample question from the questionnaire for participant group 2 is “What are your main concerns and/or challenges about implementing a therapy dog program in your school?” A sample question from the questionnaire for participant group 3 is “How are concerns/challenges raised by schools addressed?”

### Procedures

Ethics was approved for the present study by the relevant University Human Research Ethics Committee as well as the Department of Education and Training. Participants who agreed to participate in the study via signed consent forms completed the online survey and/or indicated their interest to participate in a semi-structured interview. The online survey took around 15–30 min, while the semi-structured interviews conducted over the phone varied between 20 and 90 min.

### Qualitative Methodology

The online surveys and semi-structured interviews identified participants views of therapy dog program implementation, perceived facilitative factors as well as barriers in school based setting. Thematic analysis was used to analyse all qualitative data using the framework by Clarke and Braun (59), as summarised in Table 2. Overarching themes were identified in response to the three research questions. Inter-rater reliability was established through discussions with another researcher in the research team where sections of the transcribed interviews and surveys were analysed. Both researchers discussed and reviewed the transcripts and themes together whereby 2 themes that were not in agreement and omitted as the suggested themes did not represent the quotes. The researchers drew similar conclusions to the data on all other themes and identified similar themes for the sections of transcripts and surveys. Please see Table 3 which provides an overview of the common concerns and challenges of therapy dogs in educational settings and also strategies to manage and address them.

**Table 2:** Braun and Clarke's (60) six-phase framework for thematic analysis.

**Phase**	**Description**	**Process undertaken in this study**
1. Familiarisation with the data	Transcription of data, repeated reading of the data, noting down initial ideas	All interviews were personally conducted and transcribed by the researcher. Each transcript was checked back against the original recording for accuracy and read at least three times. Early impressions of the data were noted as comments in the transcripts.
2. Generating initial codes	Organising data in a meaningful and systematic fashion across the data set	Each segment of data that was relevant to the research question was coded using a deductive approach using the PERMA framework as a key sensitising concept. A codebook was created.
3. Searching for themes	Sorting codes into potential themes	All codes were examined and collated into broader themes that address the research question. Four preliminary overarching themes were identified.
4. Reviewing the themes	Reorganisation of themes, generating a thematic map	Data was reviewed and preliminary themes were modified to ensure that they were coherent, distinct with no overlaps, and supported by data. This was done through negotiated agreement with the research team.
5. Defining and naming themes	Generating clear definitions for each theme	Main themes and subthemes were named, and a final thematic map illustrating the relationships between themes was created.
6. Producing the report	Themes and data extracts are reported and discussed	Reported and discussed themes identified in the report in relation to the research question and implications of the findings.

**Table 3:** Common concerns/challenges and strategies to manage/address them.

**Concerns**	**Recommendations**
Legal implications and liability	•Work exclusively with certified therapy dogs as both the dog and handler go through stringent training to ensure their suitability to perform the role of a therapy dog/handler team (31). Where possible, handlers should work with therapy dog organisations who have experience working with schools and are familiar with processes on implementation and common risks and concerns. •Obtain written consent from a minor child's parent/guardian to allow the child to interact with the therapy dog. Subsequently, seek child's assent on willingness to be part of the therapy dog program (27–30). •Check for the presence of any liability insurance – Dog handlers who are registered with a therapy dog organisation may be covered by liability insurance (31). •Schools are encouraged to develop a written policy to clearly identify and explain the policies and procedures of how a therapy dog will be included in the school (27).
Safety	•Certified therapy dogs are trained, rigorously evaluated, and reliably non-aggressive both to people and other dogs, highly adaptable, and interact easily with people. Dog handlers are also trained to identify signs and triggers of distress for their dogs. •Prepare students and staff for the introduction of the therapy dog and share appropriate ways of interacting with the dog (e.g., how to approach, play, feed, care for the dog, as well as how to act if they are afraid of the dog). This can be done through watching videos, modelling and role-playing appropriate behaviour, creating a schedule of when the dog is working and resting may be helpful. Students must also be trained on ways to approach and interacting with the dog (e.g., generate a list of ‘Do's and Don't's) and be given opportunities to role-model and practise appropriate behaviours. Provide potential participating children with an animal-assisted play activity group session before the program begins to assess students' suitability for interacting with dog and address or prepare for potential barriers. In the case of children with known behavioural difficulties, extra instruction in advance and extra supervision during visits may be necessary (31). •Develop safety and emergency protocols in rare instances where an accident occurs (e.g., scratches, negative dog behaviours, adverse reactions to dog). Dog handlers should immediately report them and adopt appropriate measures (e.g., removal of the dog from the situation, medical care).
Allergies	•Qualified dog handlers are typically required by their therapy dog organisations to meet certain cleanliness and grooming requirements to minimise allergic contact. For example, therapy dogs are to be bathed and well-groomed immediately before a visit to school so animal dander, the most common source of an allergic reaction, is significantly reduced. Handlers may also have anti-dander wipes on their dogs if suitable. They should also have up-to-date vaccinations and regular veterinarian checks. If a choice of dog breed is possible, consider working with dog breeds with hair rather than fur (e.g., poodles, shih-tzus, Yorkshire terriers) and who do not shed the same way as other dogs and may not cause an allergic reaction in some individuals (2). •Verify children's medical records so that children with a known allergy to dogs will not be included in the therapy dog program and steps can be taken to prevent/reduce their interactions with the dog. Parents/ guardians should be advised to consult with the child's doctor to verify if the child has any medical condition that might prohibit participation in the program, as well as to seek consultation on any preventative measures to reduce or prevent the occurrence of an allergic reaction (29). •Schools should implement clear routines for children and the handler before, during, and after interaction with the dog. For example, pre- and post-hand washing routines, regular washing of the dog's belongings, as well as pre-arranging for the dog to arrive and leave through a designated entrance after classes have begun to decrease potential contact with children (28). •Schools may consider adaptations for therapy dog programs in order to accommodate children who have allergic reactions to dogs. For example, holding a demonstration by therapy dogs in large and airy spaces or outdoors to minimise allergies in children with mild allergic reactions, or having a live event be recorded on camera and viewed at another location (e.g., on a tablet device or computer station at the school) (2, 30). •Schools should also put in place procedures on what to do if a child who was identified as not allergic to dogs, displays allergic reactions. e.g., putting in place measures like immediately removing the child from the dog, informing parents, immediate attention from the school nurse/doctor, as well as possible future plans on how the child will be re-introduced to the class/school again.
Hygiene (germs, disease, infection, parasites)	•Therapy dogs are trained not to lick or scratch, thereby controlling a major potential source of infection, as well as have regular check-ups with their vets. Dog handlers have the responsibility to ensure that the therapy dog is well-groomed, clean, have current vaccinations and veterinary checks. Responsible therapy dog handlers can provide verification of these checks upon request (2, 30). Numerous studies of health concerns about dogs have been conducted, particularly in hospital settings, where therapy dogs visit patients. The general conclusions are that: the dog is no more of a health hazard than the soles of ordinary people's shoes and that routine hand washing is the best way to address concerns (Brodie et al., 2002). If soap and water are not readily available, handlers should carry hand sanitizer or pumps can be placed on site (2). •Therapy dogs are reliably toilet trained and dog handlers should be alert to the dog's signals if it needs to go out or requires a break during the program. Dog handlers have the responsibility to clean up after their dogs such as picking up any excrement with a plastic bag and disposing it in a designated area.
	•Schools and dog handlers should employ clear measures to address sanitation concerns, for example having adults/children wash hands before and after an encounter, separating designated areas for eating and drinking, ensuring dog waste is appropriately disposed of, regularly cleaning and disinfection of areas where the dog is frequently located, as well as implementing measures if accidents occur (e.g., dog suddenly becomes ill and vomits, urinates, or defecates in school).
Animal welfare	•Schools and dog handlers should teach children the appropriate way to approach and interact with the dog, as well as keep a close watch to avoid any negative interactions with the dog. Children who lack experience with animals may react unpredictably in the presence of a dog, particularly a large dog that they may perceive as a threat. Children with behavioural difficulties may also often require closer supervision when interacting with the dogs (30). •Dog handlers are vigilant in identifying signs of injury, distress or exhaustion in their dogs and be able to respond accordingly. Suspension of the program may be required if the dog shows a negative behavioural change, fearful behaviour during interactions, or has medical concerns. •Schools and dog handlers must be mindful of overscheduling activities with the therapy dog. The Intermountain Therapy Animals professionals recommend no more than one and a half hours of work at a time for the dogs, with a 15-min break in a designated area outdoors (29). •The needs of the dog should be discussed prior to the introduction to the school and well met in the school. For example, access to food and water, exercise, breaks, as well as a safe space. The dog and handler should visit the site prior to beginning the program to be familiarised with the environment and that the dog is comfortable being around large groups of people to facilitate a smooth transition (28, 31). •The dog must never be left unsupervised when interacting with a child.
Cultural differences	•Schools should have a comprehensive understanding of its student and staff community, determine what they will accept in terms of bringing a dog to school, and approach these situations in a culturally responsive way as dogs may be perceived to be 'unclean' or ‘unsafe' in certain cultures/communities. •Identify some community organisations and agencies that might lend support on understanding this community's needs and communicating in a culturally responsive way. •Parents/staff/children of these communities must be informed of clear procedures, professional training (of both dog & handler) and type of interaction taking place so that they can make an informed decision about whether they would like their children to participate in such a program (28). •Participation in the program needs to be completely voluntary and attractive alternatives to participation must be provided (29). •In cases where children of such communities provide positive sentiments to interact with the dogs despite family's objections, schools may approach the issue sensitively with parents. Families may have a more positive perception of animals through interactions with their friends' pets, when provided opportunities to see for themselves how well-groomed and obedient the dog is, and knowledge of safety/supervision procedures that will be adopted (28).
Dislike/fear of dogs	•Avoid forcing the issue if children/staff are frightened of the dog and refuse to participate in any events involving dogs. If a child has a fear of dogs, provide means where unwanted contact with the dog can be avoided and fear can be minimised. Alternative ways for participation and learning must also be arranged for such student, such as reading from the periphery or participating remotely. •There may be instances where children who initially have a fear of dogs, through positive peer role modelling, recognise that positive encounters with dogs is possible and gradually wish to go closer to the therapy dog. Gradual contact with the therapy dog should be closely supervised (30).
Evaluation and maintenance of the program	•Monitoring and evaluation of outcomes are dependent on the intended goals of the program (e.g., reduction of stress during exams, interest in reading, classroom engagement) – appropriate and relevant tools should then be selected based on these target outcomes. This may include questionnaires/surveys (e.g., administered to measure attitudes toward reading, how enjoyable a session was, presence of positive/negative feelings), direct observations, existing school data (e.g., learning progress, attendance records), and/or interviews with students, parents, and school staff. •It is critical that students' perspectives are gathered at regular time points to ensure that any individual concerns are promptly addressed. •Dog handlers and teachers must be cognizant of students' reactions to the dogs in sessions and be prepared to provide any *ad-hoc* support and debriefing should unexpected situations occur (e.g., student is scared, does not enjoy the session). •Ongoing collaboration and communication links between dog handlers and teachers are necessary to ensure any concerns are promptly addressed and to facilitate effective implementation (e.g., discussion of materials, individual students' needs, etc.). •Measures to monitor implementation fidelity to ensure that the sessions are conducted as intended should also be embedded as part of evaluation processes. Video recording, getting another school staff who is familiar with the intended objectives of the program, or engaging a colleague who also works with therapy dogs to observe the session and provide feedback, may be helpful.

## Results

### Facilitative Factors

Results gathered from participant groups 1 and 3 indicated that the perceived facilitative factors to successful implementation of a therapy dog program in schools were: (1) flexibility of program to meet school's needs, (2) qualities of program instigator, (3) whole-school support, (4) communication, training and education, (5) considerations for dog's welfare.

#### Flexibility of Program to Meet School's Needs

Schools found that having the therapy dog program evolve flexibly and organically according to the school's needs to be beneficial to staff and students, as well as fostered a sense of belonging. The dogs had the liberty and flexibility to roam free around classrooms at school and this allowed the dog's role to be adaptable to the needs of the students whenever it was required. The therapy dogs were also said to build trust with students, which formed meaningful relationships and fostered a sense of belonging to the school for students. For example interviewee 5 suggested “*I wanted him* [the dog, Scruff] *to grow up with the kids at the school and I think that's what made it even more special is the kids, like they feel Scruff* [the dog] *is theirs. We noticed over the five years that kids who struggled to transition to the next year got better because they had a dog at school, they wanted to come to school*. *They* [the students] *sort of built up their confidence and realised that Scruff is safe and he's not going to hurt them.”* While participant 5 in the survey said: “*The* [dog-assisted reading program] *is run slightly differently at every school. We are able to adapt to most needs of the school. Such adaptations might be the time of visits.”*

#### Qualities of Program Instigator or Coordinator

The qualities of the school staff who introduced and lead the therapy dog program was identified as critical in facilitating successful program implementation. This included being emotionally aware and considerate of the school community and its diversity, such as cultural sensitivities, differing perceptions of dogs, as well as taking into account the needs of those with allergies. Being cognizant of these differences (e.g., observing children's body language, when interacting with the dog, actively seeking views and feedback), taking into account their different needs (e.g., choosing a dog breed that is hypoallergenic to meet the needs of those who have allergies to dogs), and flexibly adapting the program (e.g., not allowing the dog to roam free for certain classes).

The willingness of the program instigator to take on responsibility and be invested in the program (e.g., committing additional time and attention to set up suitable environments, multitasking classroom duties and being a dog handler), being goal-orientated (e.g., researching on the impact of therapy dogs, having a clear purpose and direction on what outcomes they would like to achieve from the program), as well as being adequately prepared (e.g., ensuring the dog is adequately trained and prepared to enter the school environment) were also factors that were highlighted. Interviewee 7: “*I did research and made sure I read some articles, and made sure we had the right blurp on the website…about the benefits of having a dog, and we made we got the right breed and called a couple of primary schools that had already implemented the therapy dog program. We made sure we had him prepared for school and for children from the beginni*ng.”

#### Whole-School Support

Participants expressed that the program was able to progress and develop with the help of parents and staff cooperation and from the backing of school staff, principal, and school council. The acceptance of the whole school community (e.g., being accepting and enthusiastic during the implementation process) facilitated the introduction of the dog to the school, as well as allowed the program instigator to be more confident in implementing the program. Interviewee 1: “*It's really that sense of community that helps*. *The parents are on board with the program*. *I think it would be very tricky to do it if the staff isn't in agreement with it*. *Being really supported by the school I think is absolutely necessary.”* Interviewee 3: “*The whole community has really got around it. The school council was definitely a support…having this you know it just affirms that I had the right idea for the community.”*

#### Communication, Education, and Training

Sharing and discussing key information on the role of a therapy dog, its possible outcomes and benefits, the training both handler and dog undergo, roles and responsibilities of school staff, risk management, as well as building students' knowledge about dogs prior to program implementation helped facilitate acceptance amongst children, parents, and school staff. Therapy dog organisations that conduct dog-assisted reading programs with schools also assign a coordinator to support the school and handlers, provide information packs and an orientation meeting to discuss key information, as well as are in regular contact with schools and handlers to obtain feedback. *Interviewee 3: “I've been very specific with the staff on this is what you need to do, this is how you need to approach so I'm constantly trying to refine that interaction and training as I go.” Survey 5: “(We) provide adequate information on our risk minimisation strategies, current insurance held, and emergency procedures… We have a coordinator assigned to support our volunteers. This coordinator organises a school orientation meeting to set up the program in a new school with a new volunteer. This meeting includes the handler and the dog, the coordinator, any teachers involved and the key liaison person for the school. The meeting works out a day, time, and place for the reading sessions. It also introduces the handler and dog to the school.”*

#### Consideration for Dog's Welfare

Ensuring that the therapy dog's needs and welfare are well-met and considered enabled them to thrive in the school setting. This included planning dog-specific and dog-friendly areas in the school, as well as scheduling down time and breaks for the dog. Interviewee 1: “*We are lucky we've got bog green ground. So instead of sitting in a classroom and talking to a child about whatever is going on, we tend to grab the dogs and take them with us for a walk around the oval.”*

### Challenges and Support Required

The following challenges and concerns were identified (1) flying solo: the workload of the instigator and handler, (2) winging it: lack of regulations, guidelines or research on implementing in schools, (3) community acceptance and buy-in, and (4) laying down foundations and acquiring therapy dog education.

#### Flying Solo: The Workload of the Instigator and Handler

Most participants were both the therapy dog handlers and instigators of the program. Difficulties faced include time constraints and added workload in addition to being a classroom staff. Participants expressed that while they were dedicated and invested in the program, they struggled with managing the responsibilities alone in the implementation process. Some participants stated that they would have appreciated other staff to be extension handlers or to take on a leadership role to share the workload of program implementation. Interviewee 5: “*I would've liked to do more but being a fulltime teacher, I just was restricted for time…and as it went on, I just got less and less support from staff involved…and the momentum stopped*.”

#### Winging It: Lack of Regulations, Guidelines or Research

Some participants stated that they had trouble finding regulations or guidelines that could facilitate implementation of their program, as well as finding little research or literature on implementing therapy dog programs into educational settings. The lack of regulations and guidelines as resources for school staff attempting to implement a program was one of the main challenges and impeding factors associated with stunted development in the implementation process. School staff expressed that without having any existing policies or guidelines to follow they had to figure out implementation on their own without any support in how to implement a therapy dog program. Interviewee 3: “*It's a bit tricky on the policy side of things because of not having anything already existing for schools, I'm sort of working a little bit from scratch…it's just even trying to understand what it should be.”* Survey 2: “*No research to share with community ideas on how to introduce the dog to the community in a positive way.”*

#### Community Acceptance and Buy-In

All participants expressed the need for the whole school community to be open and accepting of the therapy dog program. They stated that without staff support, the process would be extremely difficult. Participants expressed that school staff's and students' resistance and reservations toward the program would be a key barrier. This included barriers like managing personal views about dogs as well as risk management (e.g., allergies). Survey 1: “*The challenges would be people that maybe aren't dog people.”* Interviewee 6: “*Staff have said to me, ‘I wouldn't be happy with having a dog at the school… I don't think I would go ahead with a therapy dog unless I had a majority of buy-in from the staff.”* Survey 5: “*Increase in occurrence of a student in the school being anaphylactic to dogs, thus the program cannot start.”* Managing the rest of the students' expectations was also identified as a challenge. Survey 5: “*Every student wants to read to the dog and the majority of students in school will not get this opportunity.”*

#### Laying Down Foundations and Acquiring Therapy Dog Education

A few participants identified the need for foundational knowledge about the role of the dog in the school as well as how best to involve the dog therapeutically and effectively in school. Interviewee 6: “*I think we would need skills on how to use the dog effectively…so some sort of PD for staff of what the role and function of a therapy dog is, and probably educating staff before even looking at getting a dog, so laying a foundation.”*

### Recommendation for Therapy Dog Program Implementation

#### What Are Some Factors for Consideration Before Implementing a Therapy Dog Program?

Based on the qualitative analysis above of both the survey and interview data, as well as the literature the following factors are for consideration before implementing a therapy dog program:

##### Handler/Therapy Dog Factors

It is critical that the dogs receive appropriate certified training where they are rigorously trained and evaluated to be reliably non-aggressive to both people and other dogs regardless of circumstances, are highly adaptable, and can interact easily with people. During the training, handlers are also trained to meet welfare, safety and hygiene requirements for both the dog and students, and how to connect and engage with the dog therapeutically.

Handlers should be prepared to:

- Be personally and financially responsible for the dog's welfare and maintenance including safety, feeding, grooming, cleaning, and vaccination. In the event where the dog is involved in school programs regularly or in the long term, it is recommended for the handler to request financial support from school since the dog is part of an intervention employed for meeting the students' needs (27). In such cases, it is important to put the financial plans into a written budget outlining a list of all expenses required in order to deliver the program and share them with all responsible parties (29).
- Be vigilant in identifying signs and triggers of injury, distress, or exhaustion for their dogs and be able to respond accordingly. Regular breaks should be given to the dog. Suspension of the program may be required if the dog shows a negative behavioural change, fearful behaviour during interactions, or has medical concerns.
- Trouble-shoot when an incident occurs (e.g., when a student has a negative response to a dog), and adopt appropriate measures when needed (e.g., removal of the dog, medical care, debrief with student).

To facilitate successful implementation of therapy dog programs in schools, handlers should also have a good understanding of the impact of therapy dogs and how they may participate in various educational settings. Establishing a clear goal/purpose of the inclusion of a therapy dog in different school activities is essential – e.g., desired outcomes, who might benefit, and how (27, 31) (Freeman et al., 2016). This facilitates planning of activities (e.g., frequency and duration of activities, how the dog may be incorporated safely and appropriately, anticipated risks and concerns) as well as the evaluation of outcomes. Handlers are also encouraged to be proactive in researching on the current evidence base about therapy dogs and communicating with others who have had experience implementing such a program (29).

##### School Factors

Leadership and whole-school support is essential in successful implementation of a therapy dog program (31). The following factors on the fit and capacity of the school to undertake a therapy dog program should be considered and discussed prior to implementation:

- School staff's overall acceptance of the therapy dog – Inviting a dog into a school should not be a unilateral decision. The thoughts, concerns, and ideas of all stakeholders should be informally solicited by conducting preliminary meetings with administrators, parents, teachers and paraprofessionals and students early on to:
  ° Briefly explain the idea of involving a therapy dog in school and goals
  ° To obtain initial support
  ° Learn of any dog-related allergies or phobias
  ° Discuss and address any other concerns.
  It is recommended that a comprehensive handbook be constructed to clearly identify and explain the policies and procedures of how the dog will be included in the classroom and school activities (27).

- Stakeholders are likely to have varied concerns. Buy-in from can be facilitated by preparing information ahead of time (e.g., benefits of therapy dogs, their inclusion in schools), providing opportunities to ask questions, and preparing to respond to any potential concerns (56). Obtaining buy-in from school leaders ahead of time and presenting collaboratively to school staff is recommended (27)
- Presence of 1 or 2 other school staff who can be involved in the therapy dog program to ensure that the handler is not the sole person managing the program. This is to ensure that the work load of undertaking a therapy dog program is balanced vis-à-vis other responsibilities the handler is fronting, collaborative planning and problem-solving of programs, and implementation of effective emergency protocols if more than one person is required (e.g., handler managing the dog, other school staff who may need to contact parents or debrief with a student).
- Logistical considerations such as appropriate indoor and outdoor areas for the dog and scheduling of activities and breaks for the dog
- School-wide protocols to address any sanitation or safety concerns, which would require training school staff and students on appropriate ways to interact with the dog and the training of emergency protocols (e.g., in the event of dog scratch or bite, students' adverse reactions).
- Communication plans for engaging parents and students about the program, addressing concerns (e.g., cultural differences, fear of dogs, allergies or medical concerns), as well as obtaining consent and assent.
- Possible funding (e.g., grooming and vaccination expenses, materials required)
- Adequate planning and preparation for introducing the therapy dog to school staff so that they are educated on appropriate animal care and behavioural expectations, and are able to step in when necessary in times of emergencies (e.g., negative dog reactions in students, emergency protocols).

##### Student Factors

Assessing the needs and suitability of students whom the dog might be working with is critical in ensuring that the therapy dog program goals are met, and student welfare is considered. This includes:

- Determining which students/classes are to participate in the therapy dog program – e.g., which students would benefit the most from this program? how should the program be structured to meet their needs best (e.g., whole-class, in groups, or individually)? How does a therapy dog program value-add to existing programs in meeting the needs of these students?
- Making suitable alternatives for children who are unable to participate (e.g., for cultural or religious reasons, allergies, fear of dogs) (29)
- Deciding how expansive the program will become as it is unsurprising for many other children or families who might wish to participate after learning about the program (29)
- Adequate planning and preparation for introducing the therapy dog to students so that they are educated on appropriate animal care and behavioural expectations.

##### Parent Factors

As with engaging school staff, early engagement of parents is also essential once there is clear direction that a therapy dog program may be introduced in school. Common concerns parents have include safety, hygiene, and allergic concerns (29), how the dog will be incorporated in learning activities (27), as well as cultural differences (28). Parents must be informed of procedures and processes, be given the opportunity to ask questions and voice concerns, and provide written consent signifying they understand and support the dog's inclusion (27).

Schools are advised to ensure that there is ample time to engage parents before the commencement of a therapy dog program. This includes parents who have provided consent to their child's participation and providing further information about the program and addressing concerns, parents who do not consent and need further information on how their children will be engaged in alternative ways, as well as parents of children who are not selected to participate in the program but wish to do so (29).

## Discussion

The findings from this study highlight insights into implementing a therapy dog program in school settings, particularly a whole-school effort in optimising the program to meet unique school needs, garnering support, as well as overcoming systemic barriers. This includes ensuring the flexibility of the therapy dog program to meet varying student needs, dedication and commitment of therapy dog program coordinators/handlers, acceptance and training of all of the school staff, support from school leadership, as well as adopting a team-based rather than individual-effort in program planning and implementation.

The findings on the importance of a whole-school approach are congruent with past research. Programs are more likely to excel when they are aimed to involve the whole-school community. Research indicates that positive program outcomes are facilitated when interventions are integrated into daily practise, the school culture and encourage collaborative efforts to include staff, families, teachers, and children (61, 62). With students, this manifested as a sense of trust and connexion between the therapy dog and the school community. School staff placed importance on building foundations of trust and connexion with the students at the initial phases of implementation, as they first introduced the dog and their program to the school. With these foundations in place, the programs ran successfully and with ease, students reaped the most benefits when they formed a relationship with the dog and thus helped them connect more to the school community and have a stronger sense of belonging. A sense of belonging is also a strong indicator of a successful therapy dog program to promote well-being, which is congruent with the literature (63–65).

Another important feature is the support from leaders and a team-based approach in program implementation. Findings from this study indicated that it was mostly a single individual or program instigator/coordinator, often the dog handler and also school staff, who was solely responsible for the various stages of the program implementation, which contributed to heavy workload and potentially negative implications on the sustainability of the program. One of the key facilitative factors reported was for program instigators or coordinators to be willing to take on the diverse roles and responsibilities for successful program implementation, including acting as a promoter (66), as well as being emotionally aware and showing strong interpersonal skills as leaders of the program (67). All of which have been found to contribute to program success (39, 40). However, it appears that this has also been reported as a barrier in this study due to the high workload of these staff. Instead, taking a team-based approach and understanding that there will be a need for “multiple actors” in the implementation process is critical (39, 40).

A team-based approach to program implementation includes the support from school leaders as well as acceptance and buy-in of other school staff. Strong leadership was indicated as a strong focus for successful therapy dog program implementation, and this is also shown to be a measure of general program implementation success. Findings implied school staff needed to be prepared and goal-orientated for their program to be successful and for student reap the most benefits to their well-being. Moreover, school staff had to maintain throughout implementation a vision and a purpose, engage the whole community and constantly meeting their aims and outcomes of the program. This is congruent with the literature detailing the importance of these leadership qualities to be a role model for other staff and will provide these front-line staff with clear expectations for the program (39, 48–50).

Another key feature for successful implementation is having a strong support network from the school community, including staff, parents, principal, school council and students. School staff expressed that having the community's support and alignment of goals and purposes made introducing the program to their school easier and with fewer complications. This is congruent with the research on implementation programs requiring empowerment, participation and education of the community, and lastly, multiple actors in implementation process (obtain broad-based support of school staff) (39, 40).

A major factor that caused difficulties for school staff who had experienced implementation of a program was a lack of guidelines and regulations available for schools. The findings imply that for future practise, there needs to be further research in regulations and guidelines into therapy dog programs for schools and government support to provide policies and guidelines for schools to follow. This highlights the gap in the literature surrounding guidelines on implementation and regulations of having a therapy dog at a school. Moreover, these finding align with research on necessary implementation strategies such as government support, policy documents (68), oversight and possible regulations (39, 40).

Acknowledging the limitations of the findings of this evaluation is important. The study was small, and only the perspectives of selected school staff and therapy dog coordinators were gathered. As most of the school staff and coordinators interviewed were leaders and initiators of the therapy dog program, their feedback might be slightly biased as they had an invested interest in seeing their program be successful. As a qualitative project, the aim of data collection was not to achieve a statistically representative sample. Instead it is to attain what Glaser and Strauss call “saturation of themes” of data collection until no new themes are generated. Samples sizes have been chosen based on my extensive experience with qualitative projects of this kind (69).

Obtaining information from other school staff who may not be directly involved in the program, students, as well as parents would be beneficial to gather a more holistic understanding of having a therapy dog program in school. In addition, each school is likely to have implemented their therapy dog program in different ways (e.g., number of days the dog was in the school, how students interacted with the dog specifically); this information was not gathered in this study. Given different schools also ran their programs in different manners, potentially impacting program outcomes. It is unclear if the structure of the therapy dog program in each context could have also affected the findings. A potential limitation may be geographic/regional applicability of this study. Additional, survey participants may not have had an interactive, back-and-forth opportunity to clarify information as much as interviewees may have had in a semi-structured interview, it is unclear how this may have affected data collection. Nonetheless, this study albeit small and limited, has laid the groundwork for further research in this field.

## Conclusion

The overall findings from this evaluation highlight the facilitative factors and challenges, as well as key considerations when implementing a therapy dog program in schools, particularly the need to adopt a whole-school approach and involving multiple relevant stakeholders (e.g., handler, school leaders, school staff, students, parents) in the process. Successful implementation of therapy dogs in an education setting appear to revolve around (1) flexibility of the dog therapy program to target school's needs, (2) qualities of program instigator, (3) whole-school support, (4) communication, training and education, (5) considerations for dog's welfare. The results have also underscored the need for guidelines for schools to assess their readiness/feasibility of such a program, key factors for consideration, roles and responsibilities of key stakeholders, as well as strategies to manage challenges.

## Data Availability Statement

The raw data supporting the conclusions of this article will be made available by the authors, without undue reservation.

## Ethics Statement

The studies involving human participants were reviewed and approved by Monash University. The patients/participants provided their written informed consent to participate in this study.

## Author Contributions

All authors listed have made a substantial, direct and intellectual contribution to the work, and approved it for publication.

## Conflict of Interest

The authors declare that the research was conducted in the absence of any commercial or financial relationships that could be construed as a potential conflict of interest.
