# Supplementary material for: Therapy Dogs in Educational Settings: Guidelines and Recommendations for Implementation
Source: Front Vet Sci. 2021 Jun 8;8:655104. doi: 10.3389/fvets.2021.655104 (PMC8217446; doi:10.3389/fvets.2021.655104)
Supplement: Supplementary file 1 [file Data_Sheet_1.PDF]

APPENDIX A

Survey for school with existing programs also used as questionnaire for semi-structured interviews

Survey for Schools with an Existing Therapy Dog Program

I have read the Explanatory Statement and agree to participate in this research.

End of Block: Introduction

Start of Block: Block 4

Please provide us with the following details:

Name:

____

Designation:

____

Name of School:

____

End of Block: Block 4

Start of Block: Default Question Block

Q1. Please share the key reasons for your school's decision to implement a therapy dog program.

1a. How did your school decide on implementing a therapy dog program? (e.g., were there any specific reasons for implementing it instead of other programs?)

____

____

____

____

____

1b. How long has your school had a therapy dog program?

____

____

____

____

____

1c. Please indicate how the therapy dog and handler was prepared to participate in the therapy dog program in school.

◯ The dog was trained by the handler to be a certified therapy dog. If so, please indicate the name of the organization it is certified with. (1) ____

◯ The dog is not a certified therapy dog. If so, please briefly share how the dog and handler were trained/prepared to implement the therapy dog program in school. (2) ____

End of Block: Default Question Block

Start of Block: Block 5

Q2. Describe how therapy dogs work with students in your school.

Please tick and elaborate on the options below. Multiple responses are possible. You may use the guiding questions below to help you with your description.
What is the profile of this/these student(s) (e.g., needs, age group, background)?
What are the main goals that are hoped to be achieved with this/these student(s)?
How are the therapy dogs and their handlers included in activities with the student(s)? What is the frequency and duration of these interactions? How long do they last (e.g., over 6 weeks)?

- ▢ At the individual student level (i.e., the therapy dog works with students individually on a one-on-one basis). Please elaborate. (1) ____

- ▢ At the group level (i.e., the therapy dog works with small group(s) of students. Please elaborate. (2) ____

- ▢ At the classroom / whole-school level (i.e., the therapy dog works with entire class(es) of students). Please elaborate. (3) ____

End of Block: Block 5

Start of Block: Block 6

Q3. Describe the impact of having a therapy dog program in your school.

3a. What are the positive outcomes of the therapy dog program on students, parents, and/or teachers?

____

____

____

____

____

3b. Are there any negative outcomes associated with having the therapy dog program in the school? If yes, what are they?

____

____

____

____

____

3c. How does your school measure these outcomes? What methods do you use (e.g., questionnaires, surveys, school data, interviews, anecdotal feedback)? How frequently are outcomes measured?

____

____

____

____

____

End of Block: Block 6

Start of Block: Block 2

Q4. Describe the support and resources required for your school to implement the therapy dog program.

4a. What type of support did your school access before implementing the therapy dog program, if any (e.g., consultation with professionals, therapy dog organizations, other schools with a therapy dog program)?

____

____

____

____

____

4b. What resources did/does your school need to implement and sustain the therapy dog program each year?
Please tick and briefly describe the options below. Multiple responses are possible.

▢ Financial costs for start-up/maintenance? If yes, please share what they were/are used for and if your school accessed any funding for it. (1)
____

▢ Materials (e.g., books, furniture)? (2)
____

▢ Facilities (e.g., designated spaces)? (3)
____

▢ Manpower ((e.g., staffing requirements for supervision/management of the program, training of staff on specific skills/knowledge)? (4)
____

End of Block: Block 2

Start of Block: Block 7

Q5. Describe the key processes/steps for implementing the therapy dog program in your school.

5a. Who were the key school personnel involved in the planning, implementation, and evaluation of this program when it was first implemented? What are/were their roles and responsibilities?

____

____

____

____

____

5b. Who currently oversees the therapy program in the school? What are their roles and responsibilities? Are there any specific qualities required for such a role?

____

____

____

____

____

5c. What were the changes your school had to make to ensure that this program fits with existing school policies and practices (e.g., administrative processes, teaching programs, health and well-being policies, inclusive education policies, SOPs on hygiene and safety)?

____

____

____

____

____

5d. How has the school managed the fit of the program with diverse profiles of staff, students, and families in the school (e.g., individuals who are afraid of dogs, religious and cultural considerations)?

____

____

____

____

____

5e. How did your school communicate this program to school staff, parents, and students? What were the communication plans and key messages communicated?

____

____

____

____

____

5f. Does your school have written policy which identifies and explains policies and procedures of how a therapy dog will be included in your school?

○ Yes (1)

○ No (2)

5g. If yes, would you be open to share it with us?

○ Yes (1)

○ No (2)

End of Block: Block 7

Start of Block: Block 8

Q6. Describe any concerns/challenges that your schools have faced or is currently facing in the implementation of the therapy dog program and how they were/are addressed.

6a. What are/were some of the main concerns/challenges experienced when implementing the therapy dog program, if any?

____

____

____

____

____

6b. How are/were these concerns/challenges addressed?

____

____

____

____

____

End of Block: Block 8

Start of Block: Block 8

Q7. Describe facilitative factors that are key to implementing a therapy dog program successfully in schools.

7a. Are there any **school-related facilitative factors** that might promote successful implementation (e.g., leadership, administrative or communicative processes, school policies, teacher-related factors, environmental characteristics)?

____

____

____

____

____

7b. Are there any **student-related facilitative factors** that might play a key role in successful implementation of the program (e.g., student profile, areas of needs, preferences)?

____

____

____

____

____

7c. Are there any **parent-related facilitative factors** that might play a key role in successful implementation of the program?

____

____

____

____

____

7d. Are there any **handler or dog-related facilitative factors** that might play a key role in successful implementation of the program?

____

____

____

____

____

End of Block: Block 8

APPENDIX B

Survey for school considering a therapy dog program also used as questionnaire for semi-structured interviews

Survey for Schools Considering a Therapy Dog Program

Start of Block: Introduction

Q8 Dear Participant,

Thank you for your interest in participating in this survey for schools considering a therapy dog program.

Please take the time to read the Explanatory Statement attached in the email before you proceed with completing the survey.

By continuing on with the survey, you are providing your consent to participate.

End of Block: Introduction

Start of Block: Block 4

Q17 Please provide us with the following details:

Q18 Name:

____

Q19 Designation:

____

Q20 Name of School:

____

End of Block: Block 4

Start of Block: Default Question Block

Q1. Please share your broad goals/objectives and preliminary ideas on implementing a therapy dog program in your school.

Q1 1a. What profile of students are you targeting to be involved in the therapy dog program (e.g., their areas of needs, age group, background)?

____

____

____

____

____

Q2 1b. What are the main goals that you hope can be achieved for these students with a therapy dog program?

____

____

____

____

____

Q3 1c. Do you have any preliminary ideas on how you would you the therapy dog to be involved with students (e.g., with individual students, small groups, with a whole class) and the type of activities (e.g., literacy/language, social development)?

____

Q4 1d. What frequency and duration of the program are you considering (e.g., an hour a week)?

Q5 1e. How long are you intending to have the program for (e.g., short term over a few months, longer-term involvement which might span over a year)?

End of Block: Default Question Block

Start of Block: Block 2

Q12 Q2. Please share your concerns and the support you might require to implement a therapy dog program in your school.

Q13 2a. What are your main concerns and/or challenges about implementing a therapy dog program in your school?

____

____

____

____

____

Q14 2b. What support might you need?

____

____

____

____

____

End of Block: Block 2

Start of Block: Block 3

Q15 Would you be interested in trialing a prototype of the completed framework with your school?

◯ Yes. Please indicate the name and email of a liaison person (1) ____

◯ No. (2)

◯ Maybe next time. (3)

End of Block: Block 3
